# GPDminer: a tool for extracting named entities and analyzing relations in biological literature

**DOI:** 10.1186/s12859-024-05710-z

**Published:** 2024-03-06

**Authors:** Yeon-Ji Park, Geun-Je Yang, Chae-Bong Sohn, Soo Jun Park

**Affiliations:** 1 Department of Electronics and Communications Engineering, Kwangwoon University, 20 Gwangun-ro, Seoul, 01897 Republic of Korea; 2 Welfare & Medical ICT Research Department, Electronics and Telecommunications Research Institute, 218 Gajeong-ro, Daejeon, 34129 Republic of Korea

**Keywords:** Natural language process, Database curation, Text mining, Mining tool, Biomedical domain, Named-entity recognition, Relation extraction

## Abstract

**Purpose:**

The expansion of research across various disciplines has led to a substantial increase in published papers and journals, highlighting the necessity for reliable text mining platforms for database construction and knowledge acquisition. This abstract introduces GPDMiner(Gene, Protein, and Disease Miner), a platform designed for the biomedical domain, addressing the challenges posed by the growing volume of academic papers.

**Methods:**

GPDMiner is a text mining platform that utilizes advanced information retrieval techniques. It operates by searching PubMed for specific queries, extracting and analyzing information relevant to the biomedical field. This system is designed to discern and illustrate relationships between biomedical entities obtained from automated information extraction.

**Results:**

The implementation of GPDMiner demonstrates its efficacy in navigating the extensive corpus of biomedical literature. It efficiently retrieves, extracts, and analyzes information, highlighting significant connections between genes, proteins, and diseases. The platform also allows users to save their analytical outcomes in various formats, including Excel and images.

**Conclusion:**

GPDMiner offers a notable additional functionality among the array of text mining tools available for the biomedical field. This tool presents an effective solution for researchers to navigate and extract relevant information from the vast unstructured texts found in biomedical literature, thereby providing distinctive capabilities that set it apart from existing methodologies. Its application is expected to greatly benefit researchers in this domain, enhancing their capacity for knowledge discovery and data management.

## Introduction

The rapid advancement of contemporary research and academia has ushered in an era characterized by the astonishing production rate of new papers and academic resources [1]. While this phenomenon accelerates the dissemination of knowledge, it concurrently amplifies the challenges researchers face in maintaining and updating specialized knowledge in specific domains [2]. This issue is particularly pronounced in the field of biomedicine, attributed to the complexity of the domain and the intricate interconnectivity among various entities [3]. Consequently, researchers require considerable effort to identify and analyze pertinent information in this dynamic research environment [4]. In this context, the significance of text mining, aimed at extracting and analyzing information from vast amounts of unstructured text data, is on the ascent [5]. There is an acute demand for robust analysis in the realm of text mining, coupled with the development of platforms for database construction and knowledge acquisition [6]. While information retrieval approaches have proven efficacious for particular subjects, the continual surge in publications raises concerns regarding the efficiency of conventional search and knowledge acquisition methodologies. In biomedical research, considerable attention has been devoted to extracting information related to specific entities [7]. However, a wealth of invaluable information remains untapped within unstructured texts [8].

Most of the text mining tools available to date either focus predominantly on specific topics or fall short in processing data with intricate structures [9]. Such limitations restrict the practical applications of text mining. To address these shortcomings, in this paper, we introduce a novel text mining platform: Gene, Protein, and Disease Miner (GPDMiner). This platform is designed to automatically search, extract, and analyze the voluminous text data emerging in the field of biomedicine, enabling users to rapidly and efficiently acquire the information they seek. At the core of GPDMiner is its capability to leverage the extensive repository of papers available via PubMed. Specifically, this platform emphasizes querying specific topics from large databases and elucidating the relationships between biomedical entities in search results, thereby effectively visualizing interconnected information. Additionally, GPDMiner enhances the overall user experience by offering the ability to save and analyze results in various formats, including Excel and images.

It’s crucial to recognize that Named Entity Recognition (NER) forms an integral component of this platform. NER involves identifying specific entities, such as people, date and time information, and biological proteins, within textual data. Since the term NER was first introduced in MUC-6, there has been a growing interest in natural language processing across various domains, leading to numerous studies related to NER [10–16]. In addition, different researchers have advocated for definitions or restrictions on named entities (NE) in various ways [17, 18]. Currently, NE is classified into two categories: general NE and domain-specific NE, with our focus on the latter. NER tasks have traditionally involved rule-based approaches, unsupervised learning approaches, and feature-based supervised learning approaches [18]. However, with the rapid advancement of deep learning, new possibilities have emerged for executing NER tasks using deep learning-based approaches that effectively learn intricate and sophisticated features [19]. By integrating these cutting-edge deep learning technologies into GPDMiner, we have enabled the extraction and recognition of domain-specific entities from biomedical texts.

Such research endeavors aim to bridge the gap between information overload and knowledge discovery in the realm of biomedical research. By providing a user-friendly tool to both researchers and non-experts, GPDMiner seeks to assist individuals in effectively harnessing the wealth of information hidden within biomedical texts. This paper aims to contribute to the rapid expansion and progress of knowledge acquisition and analysis in the burgeoning field of biomedicine by examining the potential and significance of GPDMiner as a text mining platform in biomedical research.

## GPDMiner platform description

The exponential increase in research and publications across various academic disciplines underscores the importance of sophisticated and reliable platforms for text mining and knowledge acquisition [20]. In the biomedical field specifically, the complexity of numerous articles and journals presents considerable challenges for information retrieval and knowledge extraction tasks [21]. As an attempt to address these challenges, we have developed a new platform called GPDMiner by integrating novel technologies into existing research [22, 23]. By integrating statistical and dictionary-based analysis systems with Google’s Bidirectional Encoder Representations from Transformers (BERT) technology, GPDMiner offers the capability to analyze the interrelationships among genes, proteins, and diseases. The core components of GPDMiner include:

1. **Entity recognition and relationship extraction:** Utilizing deep learning models for recognizing complex biomedical entities and discerning their relationships.
2. **Data source integration:** Provides query and search functionalities from various sources, including PubMed and the US Patent database.
3. **Relationship analysis:** Extracts relationships based on the influence index of reference literature, visualizing both the union and intersection of the results.
4. **Comprehensive and reliable results:** Combining traditional statistical parsers with BERT-based learning approaches for enhanced reliability.

**Fig. 1:**
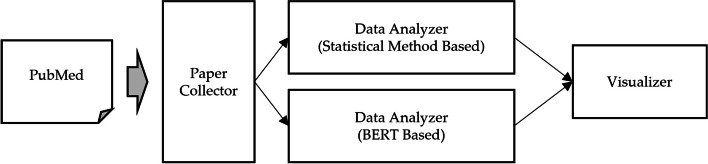
Pipeline of GPDMiner

Figure 1 provides a concise overview of the GPDMiner pipeline. Subsequent sections of this paper offer detailed descriptions of GPDMiner’s individual components and architecture, encompassing how the system collects papers, analyzes data, visualizes results, and stores them. In conclusion, GPDMiner is an innovative platform that utilizes advanced text mining techniques, allowing researchers to effectively analyze complex entities and relationships in the biomedical domain. Such capabilities present a crucial solution to address the rapidly expanding complexity and volume of data in the contemporary biomedical research environment.

### GPDMiner system architecture

In this section, we introduce the architecture of GPDMiner, a novel system for entity and relationship recognition. GPDMiner is bifurcated into a Java-based main client program and a server program equipped with a Python-based BERT artificial intelligence engine. The system utilizes the PostgreSQL database for its construction. With its capability to deliver intricate and rapid analysis in the field of biomedical sciences, GPDMiner aims to alleviate researchers’ efforts, saving time and enhancing accuracy.

**Fig. 2:**
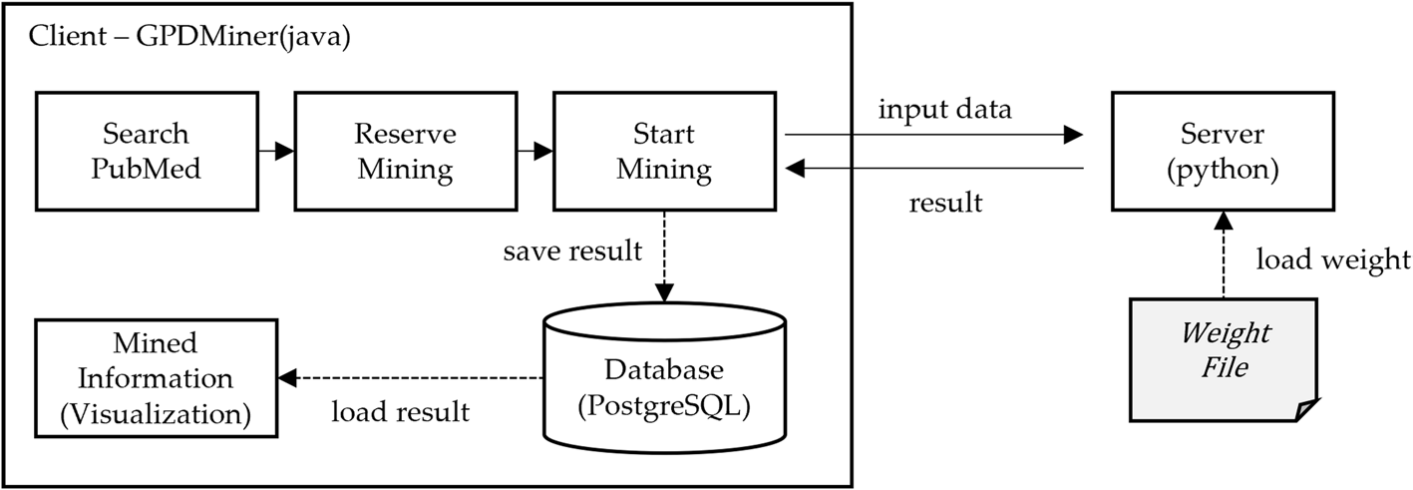
Overview of GPDMiner

The system architecture of GPDMiner is structured as follows. Figure 2 depicts the flowchart representing module-wise operations during mining execution between the GPDMiner client and the BERT server. Due to differences in programming languages, the design relies on Socket communication, transmitting data in a JSON format. This ensures seamless interaction between the client and server, enabling users to efficiently conduct PubMed searches and analyze the desired literature information.

The client searches for literature on PubMed, selects relevant publications, and transmits these data to the server using both the ’Reserve Mining’ and ’Start Mining’ functionalities. The server processes the data provided by the client by utilizing pretrained weights within the BERT server to conduct NER and Relation Extraction (RE). The server then transmits the analytical results to the client, which subsequently stores them in the database. These stored outcomes are visualized through “Mined Information,” granting users the convenience to review analytical results, thereby informing their research and decision-making processes.

**Fig. 3:**
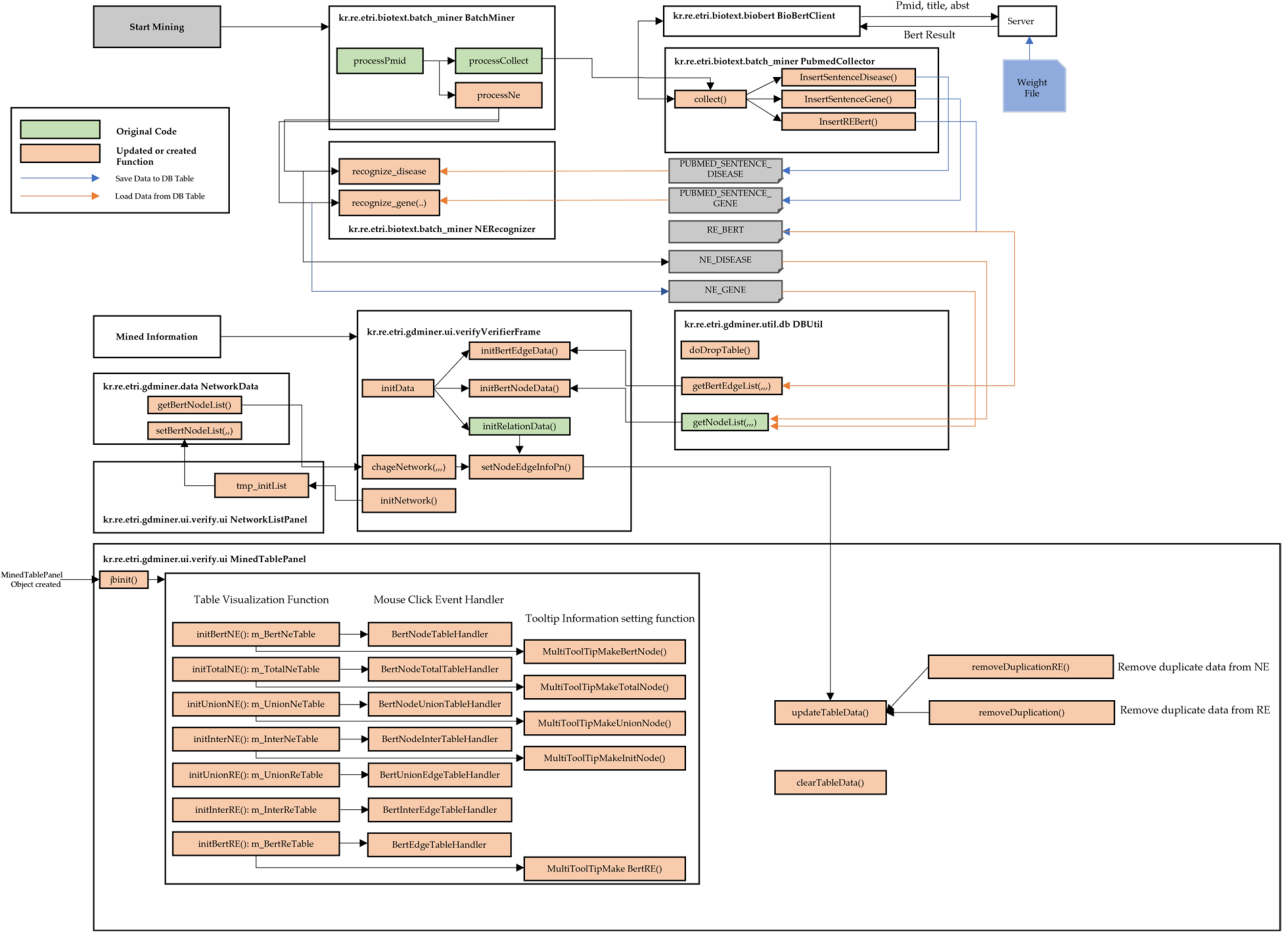
Detailed module-wise workflow of GPDMiner

Figure 3 illustrates the detailed workflow of each module in GPDMiner. This flowchart elucidates the process by which the BERT engine, integrated as a server to the preexisting client, extracts and visualizes the relationships among diseases, genes/proteins, and gene/protein-diseases for user consumption.

Within the GPDMiner client, information from selected publications is utilized to obtain NE and RE results based on both statistical methods and BERT-based approaches. These results are then stored in a database (DB) table. To visualize the stored analytical outcomes, the system generates a data list by fetching analysis data from the DB using SQL queries, based on the desired PubMed ID (PMID). Subsequent to this, data tables dedicated for visualization are produced. During this phase, any duplicate entries in these visualization tables undergo a refinement process to ensure uniqueness. Afterwards, the union and intersection data sets of the statistical method-based analysis and BERT-based analysis are generated. Through these datasets, an intricate analytical procedure ensues, culminating in the final visualization. This resultant visual representation aids users in gaining a clearer understanding of the interrelations among diseases, genes/proteins, and gene/protein-diseases.

The architecture of GPDMiner offers a streamlined analytical environment to its users. The integration of the BERT engine amplifies its capabilities, allowing for more precise relation extraction and visualization [24]. Such features hold significant value in assisting researchers and decision-making processes within the biomedical field. It’s anticipated that these functionalities will contribute to the complex data analysis and knowledge acquisition [25].

### Paper collector

GPDMiner’s search interface conforms to PubMed’s standard search methodology, with the PubMed collector playing a pivotal role. This collector employs the Entrez Programming Utilities to search for and succinctly accumulate abstract information for specific queries.

**Fig. 4:**
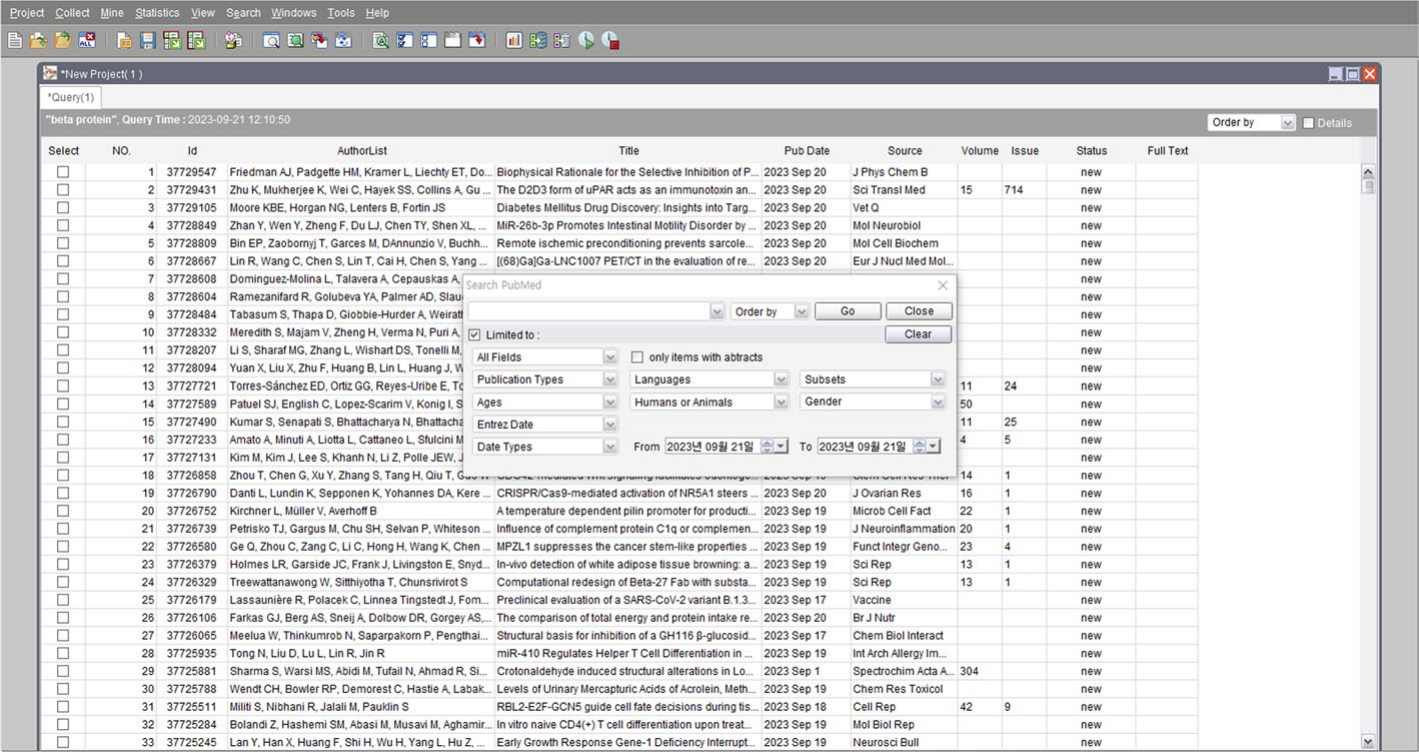
GPDMiner collector

Figure 4 depicts the search result interface of the GPDMiner Collector. Once a PubMed search is executed, the retrieved information is stored in a database. Subsequent analytical procedures extract data from this database, facilitating intricate processing tasks. Such a methodology proves crucial in efficiently (https://www.overleaf.com/project/65cf152f70616ff65e6b1f65) managing the desired abstract data, and in promptly furnishing necessary information during the analytical phase.

Users can view search results under the ’Projects’ tab. Each project comprises multiple tabs, each representing a distinct query. Within the project tab, users can review, requery, and amend the search results. Tab names are auto-generated based on the query string, although they can be renamed per user preference. Each tab displays query information, such as the query string, query timestamp, and sorting order, with the resulting abstracts presented in a tabular format. To maintain up-to-date data, either the entire list or selected items can be updated via a refresh command, with new entries automatically incorporated into the table.

This PubMed collector operates as a crucial component within the GPDMiner system, streamlining literature searches and data accumulation processes. This aids the efficiency of research and decision-making in the biomedical realm. Additionally, the flexible architecture of the GPDMiner system optimizes intricate query processing and result presentation, ensuring precise, swift access to information. The collected search and organized information are fed into GPDMiner’s Analyzer. The following sections will describe how this data is used as input for NE and RE analysis.

### Data analyzer

This section explains the functionality of GPDMiner’s Analyzer, which processes the collected data. We present results from a standard statistical-based mining approach as well as a BERT-based mining approach.

#### Statistical method based mining

The statistical mining approach undergoes the following process: Upon inputting the selected abstracts, entities and their relations are manifested through a relational analyzer. Natural language processing techniques, including Part-of-Speech (POS) tagging and syntactic parsing, are employed to facilitate this. We apply a statistical NER method underpinned by the Maximum Entropy model.

Statistical NER is a technique designed to discern named entities within a text. Among statistical techniques, we utilize the Maximum Entropy model. This model predicts which word or phrase in a given text corresponds to a particular entity type [26]. Based on principles of information theory, the model is trained to a maximum entropy state by selecting the prediction with the most uncertainty from the available data. By considering various features (e.g., words, contextual information), the model calculates the likelihood of each word or phrase belonging to a particular entity type, selecting the entity type with the highest probability. Due to its consideration of diverse features, the Maximum Entropy model can yield nuanced and flexible outcomes.

Furthermore, syntactic parsing is employed for extracting relationships between named entities. Syntactic parsing decomposes text into the structure and components of sentences, analyzing grammatical relationships between words. Through parsing, we discern roles and relations of named entities within a sentence, generating a parse tree that hierarchically represents grammatical relationships between words. Analyzing labels of each node in this tree, entities are identified and corresponding words are extracted. Relations between named entities are then deduced and extracted based on the syntactic parsing tree. By examining relationships inferred from the tree, we can comprehend the interactions between entities within the sentence.

#### BERT based mining

To attain more advanced outcomes, we present analytical findings of statistical-based mining techniques in conjunction with BERT-based mining methods.

BERT is a deep learning model grounded on the Transformer architecture, which has yielded breakthroughs in NLP. Leveraging the multi-head attention mechanism of the Transformer, BERT simultaneously accounts for the interactions of all tokens in a sentence, introducing parallel processing capabilities not seen in traditional RNN-based models. This facilitates the analysis of intricate dependencies between tokens within a sentence. The bidirectional encoding feature of BERT can concurrently capture contexts on both sides of a specific word, playing a pivotal role in entity recognition and relationship extraction. In this study, we utilize BERT’s capability to perform NE and RE in biomedical text. BERT was pre-trained with English Wikipedia and bookscorpus, but since the biomedical domain contains a variety of proper nouns and terms, BERT models aimed at understanding general-purpose languages do not perform well in biomedical test mining problems. To overcome these shortcomings, BioBERT is pre-trained using not only general texts but also medical literature collected from PubMed and PMC, which are specific corpora in the biomedical domain. We used this pre-trained model, BioBERT, to perform fine-tuning training on NE and RE tasks.

NE pertains to the process of identifying and classifying specific entities from text. In the realm of biomedical texts, this often revolves around entities such as genes, proteins, and diseases. Utilizing the BERT-based model, texts are tokenized, converted into numerical vectors, and contextually interpreted to discern the category of a given entity [27]. This procedure is integral to exacting precise information extraction from intricate biomedical contexts. RE delves into analyzing the semantic relationships between recognized entities. Bidirectional LSTMs and attention mechanisms are employed to excavate relationships between entity pairs within sentences, shedding light on complex biomedical relations. For instance, interactions between specific proteins and diseases or relations between genes and their traits are discerned. These NE and RE methodologies serve as core processes for information extraction and analysis from biomedical texts. Notably, by fine-tuning the pre-trained BERT model with a corpus tailored to the biomedical domain, we could more effectively capture domain-specific information.

For entity recognition, Fine-tuning was performed using the NCBI-Disease dataset and BC2GM dataset. Additionally, for relation recognition, the GAD dataset was employed as a fine-tuning dataset and integrated into the final relation recognition process. The detailed annotation counts for each dataset are presented in Table 1. Employing both statistical and BERT-based mining techniques, the analyzer of GPDMiner extracts vital information and discerns relationships from collected data.

**Table 1:** Statistics of datasets

Dataset	Entity type	Annotations
NCBI disease [28]	Disease	6881
BC2GM [29]	Gene/protein	13,270
GAD [30]	Gene-disease	5330

#### Mining method functionalities

In this section, we elucidate the functionalities showcasing the mining results obtained through the aforementioned techniques. To illustrate each functionality, we display the analysis results of a query for ’beta protein’ in a document with PMID 37563361.

**Fig. 5:**
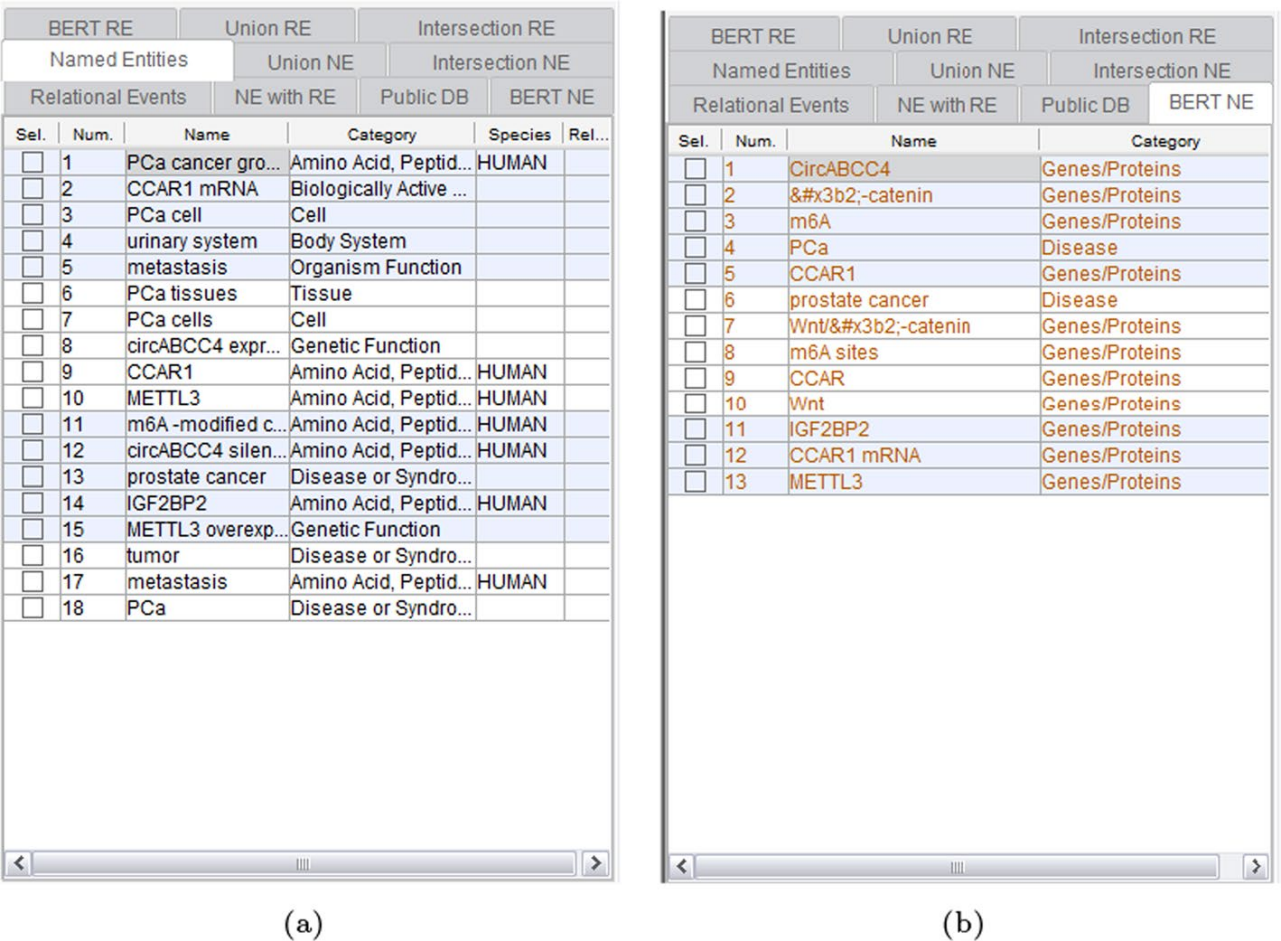
Entity recognition result of GPDMiner: **a** NER result using statistical and dictionary-based method; **b** NER result using BERT-based method

Firstly, Fig. 5 presents the results of NER by GPDMiner. Figure 5a illustrates the NER recognized solely by statistical and dictionary-based methods, which does not encompass those identified by BERT-based methods. Figure 5b highlights only the NER results identified through the BERT-based approach, distinctively marked in red to differentiate from the conventional results.

**Fig. 6:**
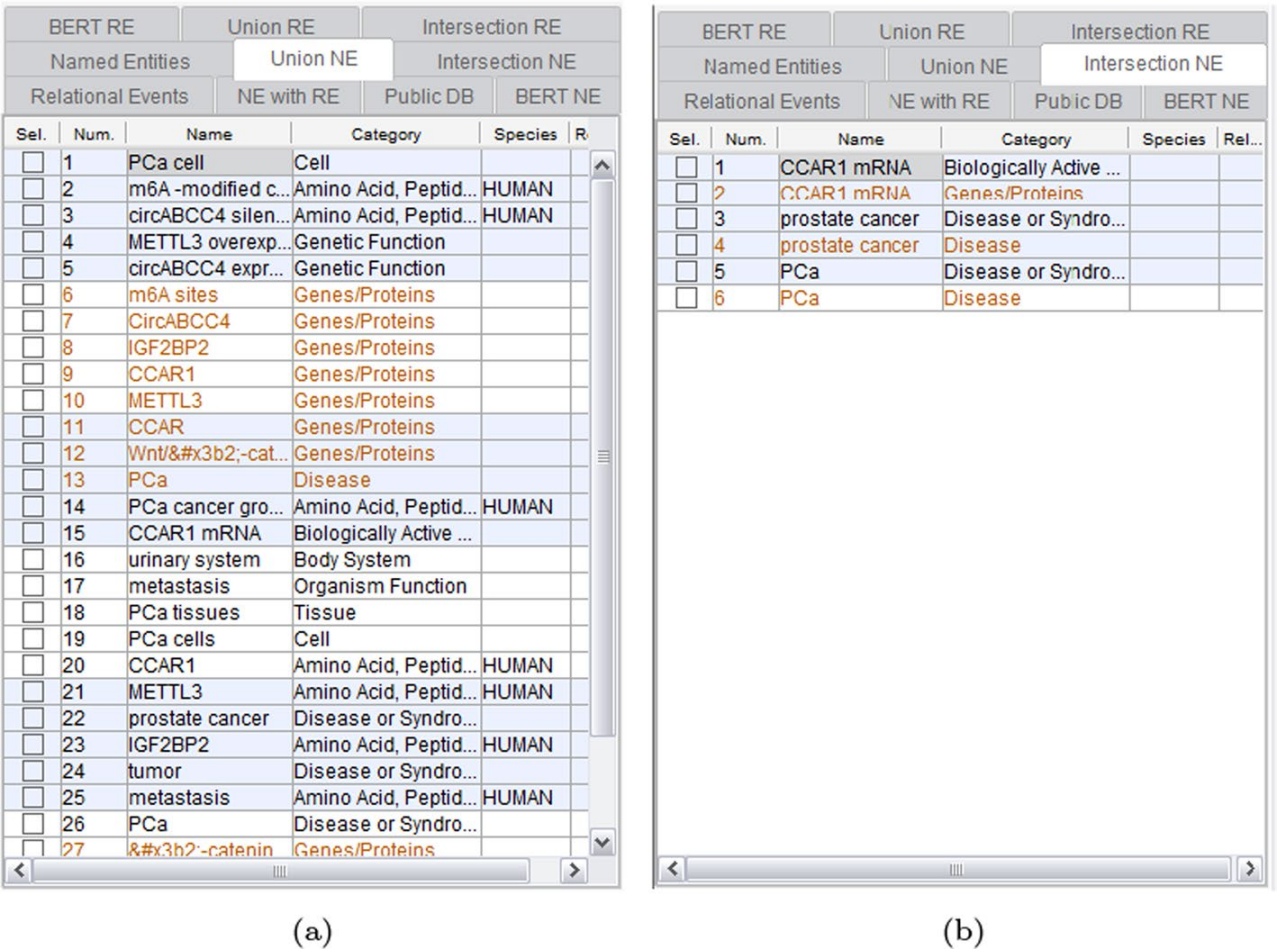
Union and intersection results applied to NER outcomes: **a** Union applied to NER results; **b** Intersection applied to NER results

Subsequently, Fig. 6 illustrates the union and intersection results applied to the NER outcomes. Figure 6a features a functionality displaying both NE recognized through conventional statistical and dictionary-based methods, as well as the BERT-based approach. Figure 6b reveals NE common to both conventional and BERT-based methods. Different colors are employed to distinguish the results obtained from the conventional and BERT-based methods.

**Fig. 7:**
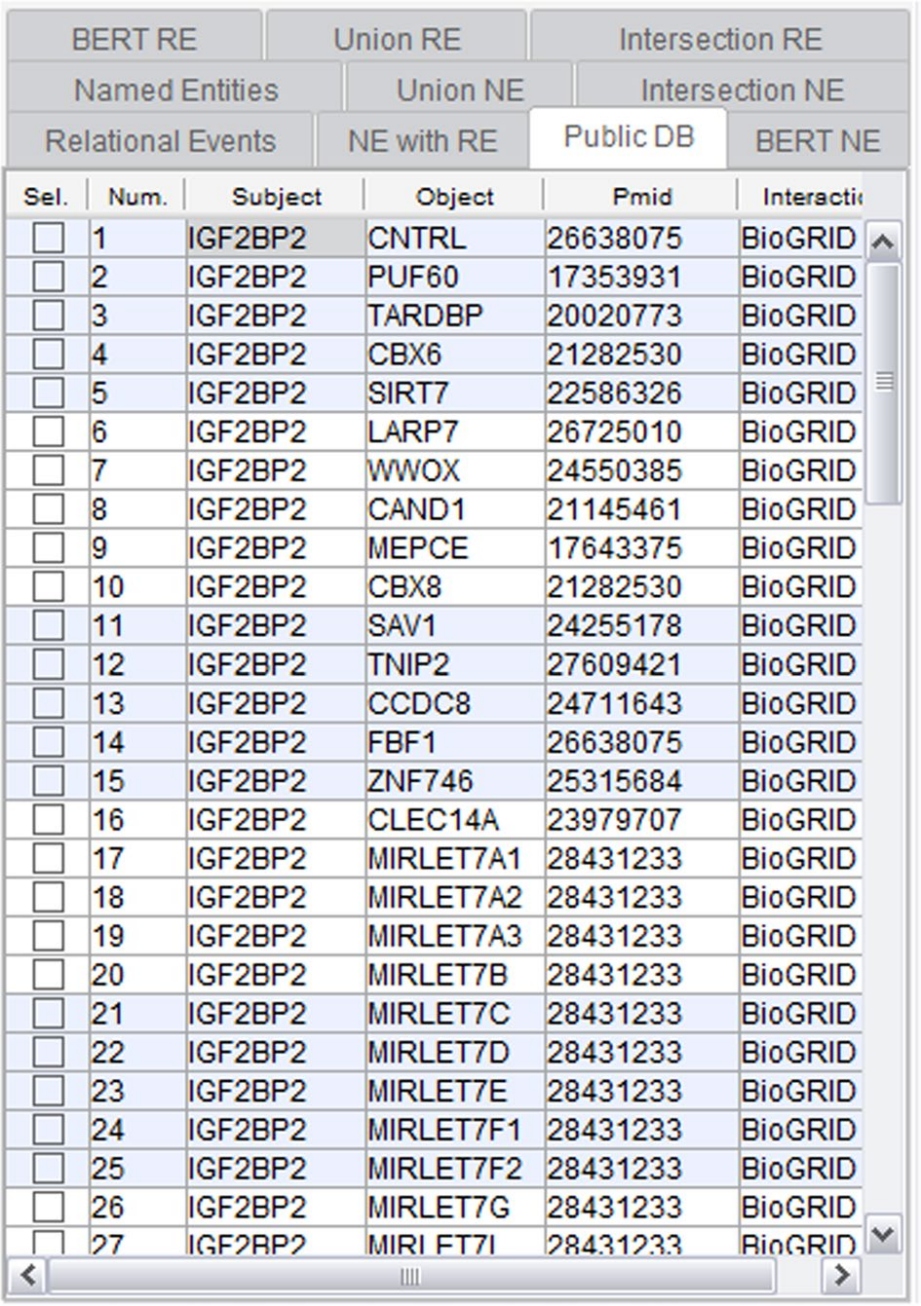
Public DB functionality

Moving forward, Fig. 7 displays the NE recognized through conventional statistical and dictionary-based methods, alongside the relevance-validated entries in public databases. This functionality provides users with relationships associated with the mined NER from public databases like HPRD and BioGRID.

**Fig. 8:**
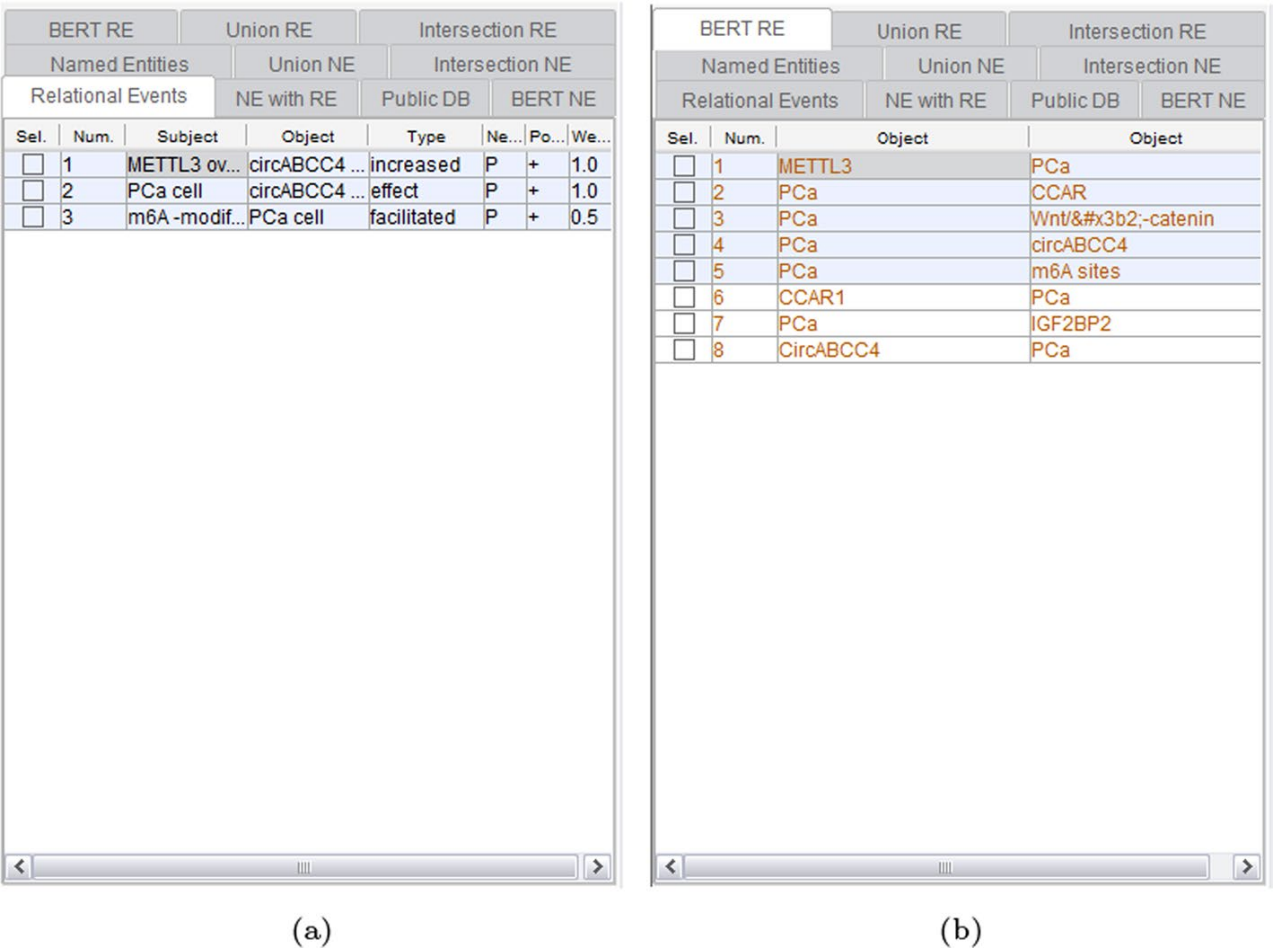
Relation extraction result of GPDMiner: **a** RE result using statistical and dictionary-based methods; **b** RE result using BERT-based method

Continuing, Fig. 8 presents the results of RE by GPDMiner. Figure 8a exhibits the relevance of entities recognized through conventional statistical and dictionary-based methods. This method displays directionality and allows for weighted display, taking into account the paper’s impact factor. Figure 8b displays the gene/protein-disease relationship extracted through the BERT-based method. However, due to the engine’s structure, the BERT-based relationship cannot show directionality and weights.

**Fig. 9:**
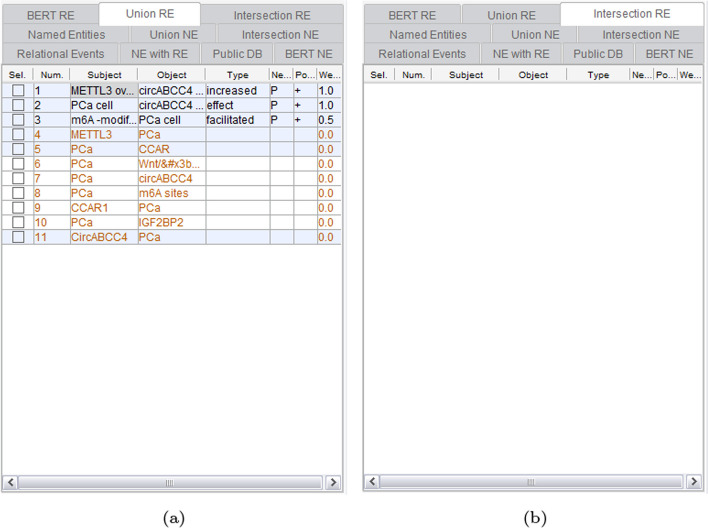
Union and intersection results applied to RE outcomes: **a** Union applied to RE results; **b** Intersection applied to RE results

Lastly, Fig. 9a comprehensively depicts the relationships extracted through both conventional and BERT-based methods. Figure 9b focuses on relationships common to both methods. The conventional results are depicted in black, while the BERT-based outcomes are highlighted in red, facilitating clear differentiation between the two approaches. This comprehensive visualization enables users to proficiently analyze and comprehend the mining findings [31].

### Visualizer

This section elucidates the process of visualizing analysis results using the data analyzer. Initiating with ’Mined Information’, the mined results of the selected project are displayed.

**Fig. 10:**
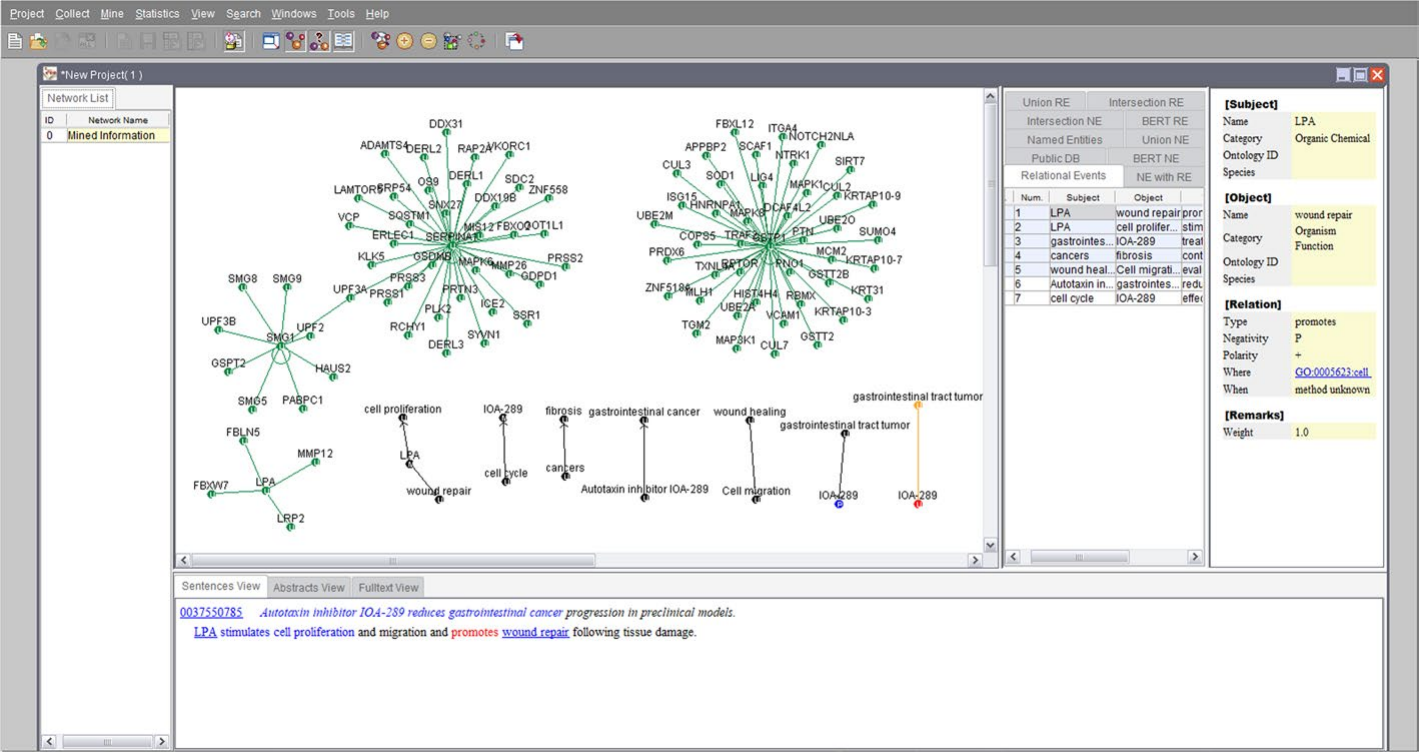
The mining analysis result interface of GPDMiner

Figure 10 illustrates the mining analysis results interface of GPDMiner. The client interface features a Network List table on the far left side, which presents a list of networks. Upon selecting a specific network, relevant information appears in the visualization screen, table screen, detailed information screen, and literature information screen on the right. The visualization screen presents data in a graphical format, while the table screen showcases NE and RE tables. By selecting specific cells in the NE and RE tables, corresponding details are presented in the detailed information screen and literature information screen. The detailed information screen presents users with a comprehensive view of an entity’s name, category, ontology ID, type, and more. In the literature information section, the selected entity/relation information is highlighted in red, while other entity/relation information is marked in blue. Clicking on a PMID connects to the PubMed site, allowing access to the abstract associated with the information.

Mining results can be visualized in this way, and Fig. 10 illustrates the ’Mined Information’ results screen for the literature with PMID 37550785, which is the result of a search query for “beta protein”. To visualize the results of the BERT analysis, we have incorporated the following.

**Fig. 11:**
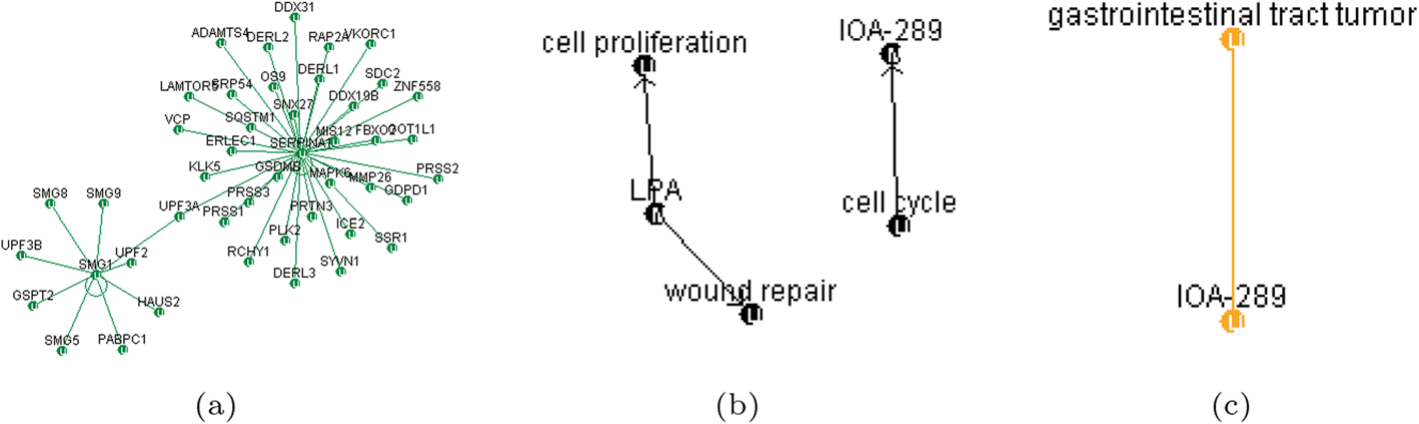
Visual representation method for RE: **a** Representation of relationships from Public DB; **b** Representation of relationships using statistical approach; **c** Representation of relationships using BERT approach

In addition to visualizing the results of relations based on Public DB and statistically derived methods, we have extended the visualization to include BERT-identified gene/protein and disease entities, as well as the relationships between gene/protein and disease. This enhancement aims to make high-quality information more readily comprehensible for users. Furthermore, to facilitate easy identification of information, we have color-coded these relationship recognition outcomes in the graphical representation. Specifically, as depicted in Fig. 11, relationships based on Public DB are denoted in green, those derived from conventional statistical and dictionary-based methods in black, and BERT-based relationships in orange.

Through the ’Mined Information’ process, users can effectively explore and interpret mining results using visualization tools and comprehensive data presentation. This user-centric interface significantly enhances the accessibility and utility of analysis outcomes, thereby supporting biomedical research and decision-making processes [32]. However, we acknowledge that more rigorous, systematic comparisons are needed to conclusively establish these benefits. Future research will focus on conducting such studies to provide empirical support for the enhanced accessibility and utility of GPDMiner.

### Saver

GPDMiner offers a suite of intuitive functionalities tailored for the efficient storage and organization of complex analytical outputs. This tool establishes a structured framework that effectively streamlines and expedites data analysis and dissemination among researchers. The ’Save Mining Report to’ feature empowers users with the capability to specify the report name, designate file pathways, and select an appropriate output format - either HTML or Excel. When opting for the Excel format, the system autonomously generates the file, encompassing three pivotal sections: ’Report’, ’Relational Events’, and ’Named Entities’. Notably, the management subsystem integrated within the ’Relational Events’ and ’Named Entities’ segments ensures an automated transition to a subsequent sheet upon reaching a threshold of 30,000 data entries per sheet. This innovative storage mechanism not only enhances efficiency but also provides the necessary scalability for handling extensive datasets, thereby reinforcing the platform’s unwavering commitment to promoting research reproducibility and transparency. While primarily designed for researchers, this feature’s utility extends to a broader demographic, offering advanced assistance for knowledge procurement and data mining endeavors, especially within the dynamic field of biomedical research where the need for efficient data management is paramount.

## Result

### Experimental setups

Table 2 shows the details of the system environment used for the experiments in this paper.

**Table 2:** Experiment environment

System environment	
CPU	Intel® Core™ i9-10920X 12-core Processor
VGA	NVIDIA GeForce RTX 3090 24GB
OS	Windows 10

The split of annotations used for training, validation, and testing on the dataset used to fine-tune the NER and RE tasks with BioBERT is shown in Table 3.

**Table 3:** Distribution of annotations

Dataset	Train	Dev	Test
NCBI disease	5130	787	960
BC2GM	5445	3060	4765
GAD	4960	0	534

The main hyper-parameters of the model utilized in GPDMiner, fine-tuned for the NER and RE tasks using these datasets, are shown in Table 4.

**Table 4:** Hyper parameters

Parameter	Value of NER	Value of RE
Epochs	30	10
Optimizer	AdamW	AdamW
Max. length	192	128
Batch size	32	32
Learning rate	0.00005	0.00002

### Experimental results

For the evaluation of our system, we performed a feature comparison with other text mining tools. One of the tools we compared against is the GENIA Tagger [33], a versatile tool encompassing morphological tagging, shallow parsing, and named entity recognition specialized for biomedical text. It has been meticulously trained on a diverse set of corpora, including the Wall Street Journal corpus, GENIA corpus, PennBioIE corpus, and NLPBA dataset for named entity recognition. Another noteworthy contender is tmTool [34], a web-based text mining utility equipped with batch processing capabilities. It excels in processing raw text from a wide spectrum of biomedical sources, including literature, patents, and medical records. tmTool integrates several entity tagging systems such as tmChem [35], DNorm [36], GNormPlus [37], and tmVar [38], enhancing its adaptability. We also examined PubTerm [39], a web-based tool tailored to support the analysis, annotation, and curation of biomedical scientific literature-a task integral to biomedical research, database curation, and clinical practice. EzTag, a web-based annotation tool, was another contender in our comparative evaluation. It empowers curators to annotate various biological concepts using pre-trained annotators like GNormPlus [37], tmVar [38], and TaggerOne [40]. Notably, it offers the flexibility to perform annotation tasks with or without existing training data, as well as the manual generation of training data. Lastly, TeamTat, a web-based annotation platform, offers efficient project management capabilities for collaborative annotation projects. It prioritizes project management features, facilitating multi-user annotation, figure display, and overall project coordination.

To gauge the performance of our NER and RE models, we employed standard metrics, namely precision, recall, and F1 score. In Table 5, you can find a comparative analysis of our fine-tuned NER model, tailored to our experimental environment, alongside NER models utilized in other systems. This evaluation provides insights into the efficacy of our system in comparison to established alternatives.

**Table 5:** Comparison of NER models for genes/proteins, diseases

Entity types	Models	Precision	Recall	F1-score
	Ours	0.8933	0.9300	0.9112
	TaggerOne Joint [40]	0.8510	0.8080	0.8290
	TaggerOne NER-only [40]	0.8350	0.7960	0.8150
Disease	DNorm [36]	0.8030	0.7630	0.8720
	Sachan et al. [41]	0.8641	0.8831	0.8734
	CollaboNet [42]	0.8548	0.8727	0.8636
	LSTM-CRF (iii) of Habibi et al. [43]	0.8531	0.8358	0.8444
	Ours	0.8551	0.8899	0.8719
	GNormPlus [37]	0.7840	0.7920	0.7880
Gene/protein	Sachan et al. [41]	0.8181	0.8157	0.8169
	CollaboNet [42]	0.8049	0.7899	0.7973
	LSTM-CRF (iii) of Habibi et al. [43]	0.7750	0.7813	0.7782

Experiments with the fine-tuned model in the gene/protein category show the best performance, with precision around 89%, recall around 93%, and F1-score around 91%. This compares favorably with other studies. In particular, other text mining tools such as ezTag, tmTool, and PubTerm use the GNormPlus model, which performs about 79% in terms of F1-score. In the disease category, we also experimented with BioBERT, which performs well among competing models with an F1-score of about 86%. Here, ezTag uses the TaggerOne model, while the tmTool and PubTerm tools use the DNorm model. The F1-scores of the TaggerOne and DNorm models are about 83% and 87%, respectively, which shows that our fine-tuned model is quite competitive in disease recognition.

For the RE task, the experimental results of the fine-tuned model show a precision of about 76.65%, a recall of about 91.1%, and an F1-score of 83.25%. There are no other text mining tools that provide RE results, making it difficult to compare performance on RE, which suggests that our system is unique for the RE task.

As shown in Table 6, both our system and other text mining tools provide built-in NER capabilities for disease and gene research domains. Unlike other text mining tools, our system has the unique ability to visualize relationship extraction results. This is a feature not found in other tools and is designed to help users intuitively understand and analyze complex relationships between recognized entities. This comparison gives us a clear picture of the strengths and weaknesses of each tool and can help you choose the best one for your specific research purposes.

**Table 6:** Function comparison with text mining tools

Tool	NER	Collaboration	RE
Ours	O	X	O
EzTag	O	X	X
TeamTat	O	O	X
GENIA tagger	O	X	X
TmTool	O	X	X
PubTerm	O	X	X

## Conclusions and future work

In this study, we introduce GPDMiner, an integrated data mining platform that innovatively supports the complex data analyses within the field of biomedicine. This system offers an amalgamation of diverse methodologies coupled with a user-centric interface, thereby mitigating the intricacies of data analysis while enhancing accuracy. A defining feature of GPDMiner is its integration of BERT-based NER and RE, along with a plethora of visualization functionalities. This allows for the intuitive comprehension of intricate analytical outputs. Advanced features for result storage and management facilitate efficient handling of large-scale data, further promoting research reproducibility and transparency. In response to the concerns raised in the review about the sufficiency of our datasets for fine-tuning our BERT-based model, we plan to develop a tagging tool system for augmenting our datasets through additional tagging. This will enable us to retrain our model with a more robust and comprehensive dataset, leading to significant performance improvements. The system we have currently implemented will serve as the base for this more advanced, final system that we plan to develop. The decision to not focus on performance analysis in this study is informed by the common observation that conventional text mining systems often exhibit significant performance degradation when deployed in real-world scenarios. Given GPDMiner’s seamless operation in real-world settings without performance degradation, we find it unnecessary to conduct performance comparisons with other systems. Our primary objective is to provide a system that is easy to use and aids researchers and experts in their data analysis endeavors within the biomedical field. Future research directions will emphasize improvements in algorithmic performance and user experience optimization. By expanding the current functionalities and enhancing the intuitive nature of the interface, we aim to better support researchers and experts in more effectively analyzing and interpreting data. Through the continual integration of cutting-edge technologies and recent research findings, GPDMiner is poised to set new standards in data analysis within the realm of biomedicine. GPDMiner represents an important step forward for data mining in biomedical sciences, simplifying complex analytical processes and improving researchers’ work efficiency. Future research is projected to broaden GPDMiner’s functionalities and increase its efficacy to further drive its significance in research and decision-making in this area.

## Data Availability

All data generated or analyzed during this study are available upon reasonable request from the corresponding author.
